# The long-term oral health consequences of an amalgam phase-out

**DOI:** 10.1038/s41415-024-7992-5

**Published:** 2025-04-25

**Authors:** Oliver Bailey

**Affiliations:** Clinical Fellow, School of Dental Sciences, Newcastle University, Newcastle upon Tyne, United Kingdom

## Abstract

Understanding the long-term oral health implications of an amalgam phase-out is complex. However, amalgam is a simpler, cheaper, quicker, more predictable and effective material to place and replace than composite, which is the main alternative. It also has fewer postoperative complications in United Kingdom (UK) primary care and has been shown to be more cost-effective over a lifetime. Existing economic evaluations are limited, however, with rudimentary models which fail to consider clinicians and patients, and likely significantly underestimate the broader costs of placing composite compared to amalgam. Amalgam alternatives require improvement and their environmental impacts require characterisation. Composite restorations can be successful in extensive cavities, but they require much technical skill and expensive and time-consuming specialised equipment, which are not being commonly used in UK primary care, especially by National Health Service (NHS) dentists. Postgraduate composite education is not generally making UK clinicians confident when faced with difficult cavities and requires improvement. Expert consensus on the use of techniques to restore varying cavity presentations with composite would help to guide this, while also considering how its dissemination could be improved. NHS clinician fees are significantly lower than in Europe. The NHS system therefore essentially incentives the use of amalgam and disincentivises the use of expensive and time-consuming recommended equipment for composite restorations. This has likely contributed to a failure of clinicians to upskill and be confident in providing posterior composite restorations safely. These issues, alongside a loss of trust, have led to dentists leaving the NHS, which has created access issues for patients, disproportionately affecting the most at need in society. An amalgam phase-out would almost certainly exacerbate this issue, widening existing health inequalities while not providing restoration characteristics which the most affected patients most value. Failure to urgently address these issues risks an oral health crisis in the UK if amalgam is imminently phased out.

## Key points

- Phasing out amalgam presents complex challenges due to its cost-effectiveness and quicker and simpler placement compared to composite, which requires more technical skill and time-consuming expensive equipment to place predictably and effectively.
- The NHS remuneration system, which favours amalgam use by providing significantly lower fees compared to the rest of Europe, is likely contributing to a failure of dentists to upskill or use recommended time-consuming and expensive equipment, which would allow them to place composite restorations safely.
- Dentists are leaving the NHS, creating access issues, which disproportionately affects those most at need in society while widening existing oral health inequalities and this would likely be exacerbated by an amalgam phase-out.

## Introduction

Amalgam use has been phased-down and will be phased-out in the European Union (EU) in 2025.^1^ The United Kingdom (UK) is assessing the feasibility of a complete phase-out by 2030 following the 2017 phase-down.^2^ This is based on the Minamata Convention on Mercury, which is a global treaty designed to protect the environment and human health by limiting the use of mercury.^3^ Most countries in the EU either already have an amalgam ban, or use relatively small numbers of amalgam restorations.^4^ The UK is different in that, especially under National Health Service (NHS) provision, posterior teeth are much more commonly restored with amalgam.^5^^,^^6^^,^^7^ NHS dental care is publicly funded but with co-payments for many.

## Alternatives

Composite resin was described as the only reasonable alternative to amalgam in the proposed timeframe for the phase-down and phase-out of amalgam.^8^ A more recent World Dental Federation (FDI)-approved review reported recent evidence to suggest that glass ionomer cements (GICs) and their derivatives may be valid alternatives for small cavities, though follow-up is limited.^9^ The review focused on direct composite, however, and concluded that there is no single material which can replace amalgam in all applications. It also noted that amalgam is favoured in health systems with limited resources due to the higher costs of the alternatives. The review's discussion centred on difficult situations. These included restoring teeth where cavity margins are deep sub-gingival, caries risk is high (for example, in the older person and those of low socioeconomic status) and cooperation is limited, as seen in patients with disabilities. The review also noted the need to improve the alternative materials' properties and demonstrate their clinical performance, especially in ‘real-world settings and for special risk groups'. Indirect restorations will be briefly discussed later.

## Direct restoration survival

Restoration survival is hugely complex and multifactorial, and the material used, though often important, is just one of many relevant factors. In a recent review investigating the survival of composite restorations (ie not including amalgam), the operator, compromise of the tooth (number of surfaces involved in a cavity and presence of a root filling, for example) and the patient and their risk factors (caries, parafunction and socioeconomic status, for example) were much more important than the material.^10^ This did, however, come with the caveat ‘assuming that materials and techniques are properly applied by dentists'.^10^ This may be a significant issue in primary care and more important for composite than amalgam restorations, as will be explored. When attempting to understand the implications of an amalgam phase-out, it is necessary to understand that they will be affected by these variables and many others, including societal norms, healthcare systems and the prevalence of caries in the population, alongside clinicians appropriately implementing prevention, non-operative and operative intervention, and reintervention. There is limited evidence suggesting that UK primary care clinicians are often not managing caries appropriately due to multiple complex factors, which need to be addressed. ^7^^,^^11^^,^^12^^,^^13^ This paper will, however, now primarily focus on the impact of the direct restorative materials.

## Differences between amalgam and composite

This paper aims to review and synthesise the existing evidence base relating to the long-term oral health consequences of an amalgam phase-out. To understand these consequences, it is necessary to understand the differences between amalgam and composite restorations. Studies tend to focus narrowly on restoration survival, but the materials vary broadly in other ways, which are important to patients, clinicians and funders. These factors can affect uptake of treatment and access to care, which can indirectly affect oral health consequences. These consequences cannot be divorced from a consideration of the differing costs in any healthcare system with limited funding, as this can affect outcomes and are an important factor in their own right. The following narrative review will therefore outline the differences in materials, discussing their clinical outcomes and how the differences in restorative processes involved can affect these. It will explore how these, in turn, can be affected by the setting in which they are provided and the costs involved. The review will critically evaluate the economic evaluation data comparing the materials and consider funder, patient and clinician perspectives before addressing who a phase-out will likely most affect in society. It will explore how all of these elements can affect long-term health outcomes, relating this to an NHS primary care context. Previously proposed future goals will be critically appraised and suggestions made.

## Clinical outcomes

The relative clinical outcomes between amalgam and composite are often fiercely contested,^14^ with a balanced discussion of the evidence base rarely taken. Relevant clinical outcomes include postoperative issues, such as sensitivity and food packing, restoration survival, failure mode and mode of reintervention, which might ultimately relate to tooth survival. As an example, those supporting the use of composite will nearly always cite one study which shows superior survival of composite over amalgam.^15^ Amalgam survival was higher in high caries-risk patients, however, and while all of these differences were statistically significant, there was minimal clinical difference in outcomes. The mean annual failure rates (AFRs) were very low for extensive (three surfaces or more) restorations with long-term follow-up. Not commonly mentioned is that this retrospective data comes from treatment by a single expert specialist Dutch dentist, who frequently used multiple matrices per tooth (as per a personal communication with N. Opdam in 2024), and that composite restorations with GIC liners were excluded, which were likely deeper restorations. This, therefore, makes the direct comparison of the materials questionable and translation of this data to NHS primary care inappropriate.

## Restoration survival

A Cochrane review which included only randomised controlled trials in two meta-analyses suggests that posterior amalgam restorations survive longer than composite with large differences.^16^ Again, there are issues which can be levelled at this data, the main one being that data in the primary analysis mostly involved children who are often at high caries risk. The data in the secondary analysis did not, however, but came from split-mouth studies with smaller numbers of restorations. It showed similar results, but with slightly reduced effect sizes. The studies are not particularly recent and resin-based materials and techniques have likely improved since then. There have been many non-controlled studies published, but they are generally at significant risk of bias against amalgam. This was explicitly shown in a large Norwegian prospective study where clinicians favoured the use of amalgam over composite in difficult situations (relating to high caries risk, lesion depth and tooth type).^17^ Despite this, the AFR was significantly lower in amalgam than composite, but both AFRs were low.^18^ This phenomenon, of choosing one intervention over another based on circumstances, is termed ‘indication bias' and is an issue with nearly all non-controlled data. This makes the drawing of comparisons in these studies problematic. There is minimal data in the UK comparing amalgam and composite posterior restorations. One practice-based cross-sectional study was published in 1999, concluding that amalgam provided significantly greater longevity than composite in posterior restorations, but the study used a potentially misleading metric to estimate restoration performance, which has been discounted.^19^^,^^20^

## Postoperative complications

Though a Cochrane review noted a difference in postoperative sensitivity favouring composite over amalgam at one point in time in the only included study, it did not consider this to be clinically relevant.^16^ These data are in stark contrast to UK primary care clinician-reported data, which showed significantly increased chances of postoperative complications, such as sensitivity and food packing with composite compared to amalgam.^7^ Here, 42% reported food packing and 46% sensitivity when placing composites in more than 10% of restorations, compared to 14% and 18% with amalgam, respectively. Additionally, 13% reported food packing and 17% sensitivity in more than 25% of composite restorations compared to 3% and 4% with amalgam, respectively. Private dentists reported the lowest incidence of sensitivity and food packing following direct composite placement compared to other clinicians, whereas 15% of dental therapists reported sensitivity in more than 50% of composite restorations placed. This could well have influenced their relatively reduced likelihood of confidence compared to other primary care practitioners when placing composite restorations and highlights potential issues of therapist education in the UK as they become an expanding part of the workforce.^7^^,^^21^

## Failure modes

The major ways in which direct posterior restorations fail are caries associated with restorations (CARS) (previously referred to as recurrent or secondary caries), followed by fracture (of the tooth and/or restoration) and then pulpal or endodontic complications.^19^^,^^22^^,^^23^ Composite restorations are more at risk of CARS than amalgam but evidence for differences between the materials in other failure modes is contradictory and uncertain, and failure mode can vary over time.^15^^,^^16^^,^^19^^,^^22^^,^^23^^,^^24^^,^^25^

These general differences in clinical outcomes between the materials are likely primarily because composite restorations are technically more difficult to perform, especially in difficult situations, though some other issues are relevant.^21^^,^^26^^,^^27^

## Caries associated with restorations

CARS detection methods are poorly validated and pose significant diagnostic difficulties.^28^ Differentiation of non-carious staining of restorative margins from CARS is difficult in clinical studies, especially with composite compared to amalgam, potentially resulting in premature re-intervention.^22^^,^^29^ CARS most commonly occurs (>90%) at the gingival margin of restorations,^28^ which likely then makes the subsequent re-restoration more difficult to perform, and especially so with composite.

CARS can be associated with a defective restoration which allows the sheltered accumulation of biofilm. This can result from ledged restorations but is likely primarily due to gaps between the restoration and the cavity wall.^28^ The likelihood of peripheral gaps between the tooth and restoration is much increased with composite compared to amalgam for several reasons, making the placement process much more technique-sensitive. This can also preferentially predispose composite to postoperative sensitivity. Composite materials may also favour a more cariogenic biofilm accumulation compared to amalgam, potentially predisposing them to CARS.^30^^,^^31^

## Fractures

In restored teeth, the restoration and/or teeth may fracture. Data suggests that 77% of tooth fractures are associated with teeth having three or more surfaces restored, and vital teeth suffer more favourable supra-gingival fractures (91%) than non-vital teeth (61%).^32^ Expert guidance recommends indirect cuspal coverage restorations for posterior root canal-treated (RCT) teeth generally, and vital teeth with biomechanical compromise, to reduce fracture risk.^33^^,^^34^ These restorations are much more costly and time-consuming to perform than direct restorations, however, and were often not provided for RCT teeth in UK primary care, likely due to the higher cost.^35^ In the NHS setting, though reintervention rates for crowned teeth were lower than for directly restored teeth, tooth survival was reduced.^36^ These data are at high risk of indication bias, however, as indirect restorations are likely performed on more broken-down teeth. It is also old. No equivalent data has been available since 2006 due to the change in NHS remuneration.

## Direct material differences which could affect tooth fracture

Cavity preparations advised for the two materials commonly vary based on how they are retained. Amalgam preparations are commonly more box-like, closed and upright, with preparation of sound tooth structure to provide mechanical undercuts, whereas composite preparations commonly have more flare and are open and saucer-shaped, not requiring mechanical retention form due to the adhesion obtained. Composite preparations are therefore purportedly more minimally invasive.^11^^,^^27^^,^^37^ However, one large prospective practice-based study showed that more conventional (amalgam-like)-shaped preparations performed better in terms of composite restoration survival than saucer-shaped preparations when controlling for many other potentially relevant factors, including the operator.^18^

Countering this data, it might, however, be expected that the (slightly) more destructive amalgam preparations would result in a higher prevalence of tooth fractures. This may especially be so given that amalgam is generally then not bonded to the remaining tooth, therefore failing to recover lost stiffness of the restored tooth unit in comparison to composite. Some laboratory studies support this whereas others do not, showing more favourable failure of amalgam-restored teeth in certain situations.^38^ One study, with previously highlighted methodological issues, showed a small increased likelihood of tooth fracture in amalgam compared to composite restored teeth.^15^ A Cochrane review and other, large clinical data do not, however.^14^^,^^16^^,^^39^ Though people commonly say that they see more tooth fractures associated with amalgam restorations (higher incidence), and this is likely correct, they commonly come to an unjustifiable conclusion that amalgam-restored teeth have a higher rate of fracture (prevalence). They are not considering the relative number of amalgam-to-composite-restored teeth that they see, suffering from a ‘narrative fallacy' and ‘base case neglect'.^39^ Many more amalgam restorations were present in a large sample where such data were collected looking at fracture prevalence. There was no significant difference between the materials (with a slightly increased fracture rate associated with composite restorations). The study had limitations in that it was cross-sectional, with no knowledge of the preparations performed, restorations' ages or relative sizes.^39^

## Restorative process differences

Amalgam is compacted under firm pressure during its application into a cavity and undergoes a very small expansion, both of which favour marginal adaptation and avoidance of gaps. In contrast, composite shrinks on setting and is more difficult to adapt during placement due to its softer consistency. It also commonly needs to be placed in multiple increments to respect depth of cure and reduce damaging contraction stress, which again increases the chance of gap formation.^40^ These issues can also contribute to the increased failure to form contact points with composite when not using specialised equipment, which can potentially contribute to material fracture, food packing and CARS.^7^^,^^26^

The effective application of a bonding agent to the tooth is required to prevent composite from pulling away from the cavity walls during polymerisation. Achieving an effective bond can be affected by many things, including the tooth substrate type (enamel or dentine) and its disease-affected state, the bonding agent composition and application, and contamination, which therefore requires the cavity to be meticulously isolated from the oral environment (ideally with a rubber dam [RD]).^31^^,^^41^^,^^42^ This can be especially challenging where cavity margins are sub-gingival.^43^ Incomplete light-curing of composite at the base of a cavity can occur without attention to detail and can result in washout of uncured components.^28^ Gaps can form following degradation of the composite bond over time, which occurs especially with dentine margins.^31^^,^^41^

The integrity of an amalgam restoration does not depend on these things to obtain a marginal seal. The technical process is much more complex when providing a composite compared to an amalgam restoration. Many of these issues can, however, potentially be overcome with appropriate materials and techniques, which will be discussed, and as demonstrated by high comparative success rates in specific but limited studies.^15^^,^^44^

Posterior composite restorations take significantly longer than amalgam to place.^5^^,^^7^^,^^45^ There is a huge array of materials and equipment which can be used to place composite restorations, with a large majority of UK primary care clinicians feeling there was a lack of consensus on which materials and techniques to use.^21^ Evidence-based guidance on placement of posterior composites advises the use of relatively expensive equipment, such as sectional matrix systems and RD.^46^ They are rarely used in UK primary care, however,^7^ especially by primarily NHS compared to private practitioners (Appendix 1). This equipment offers improved outcomes, minimising postoperative complications which are highly valued by patients, but takes longer and can be technically difficult to place.^7^^,^^26^^,^^29^^,^^43^^,^^47^ In some health systems, a fee is chargeable for placing a RD, clearly trying to incentivise the use of recommended techniques to optimise outcomes.^48^ Whereas Class I cavities vary minimally in their presentation, Class II cavities can have huge variation, which can influence the technical aspects of restoration, especially for composite. As more tooth structure is lost and margins extend deeper sub-gingivally, placing a well-adapted matrix-wedge (sometimes with an added separating ring) assembly to directly restore a tooth becomes much more challenging.^43^ Because the marginal seal is not as critical for amalgam, they are often favoured in these more difficult situations.^6^^,^^49^

The cavity variables are often not considered in most randomised controlled trials involving composite, which tend to focus on comparing materials. Studies generally include the treatment of simple cavities with low-risk patients by experts, who commonly use specialised equipment without time constraints.^25^ Follow-up is commonly limited and AFRs are therefore often very low. These studies are not translatable to primary care where all patients, whatever their risk, and all cavities have to be treated.^25^ Some expert-led opinion papers offer technical guidance on placing composite restorations in varying and difficult situations,^26^^,^^43^^,^^50^^,^^51^^,^^52^^,^^53^ but there is no real clinical evidence base or expert consensus to draw on in terms of how varying techniques influence outcomes when restoring varying cavity presentations. Modern techniques, such as injection moulding with bulk-fill composites and simplified sectional matrix techniques without the use of a separating ring, can offer simpler, more predictable and efficient solutions.^26^^,^^40^^,^^54^^,^^55^^,^^56^

## Differences in confidence

Though a large majority of UK primary care clinicians were confident placing posterior composites in standard situations, 67% from a sample of over 1,500 reported no or low confidence placing composite in patients with limited cooperation, compared to just 7% with amalgam.^21^ Similarly, 51% reported low or no confidence when restoring sub-gingival cavities with composite, compared to just 4% with amalgam. This was despite a large majority having attended postgraduate composite courses.^21^ This suggests a failure of education given the publication of articles offering guidance on using composite in sub-gingival cavities.^43^^,^^50^^,^^51^ These journal articles are often not easily accessible to primary care clinicians however. Expert consensus guidance on restoration technique may help but disseminating guidance to primary care is a challenge with multiple barriers.^13^

## Undergraduate to primary care transition

The vast majority of new UK graduates move from a university environment where they predominantly use composite, into a foundational training year under NHS provision where they commonly favour and place more amalgams.^7^^,^^49^^,^^57^ Most UK dentists are primarily NHS practitioners in the first five years following qualification and composite use increases as a clinician's number of years qualified increases,^7^ which is an opposite trend to that seen in Australia, for example, where private practice predominates.^58^

## Composite skill development

Among UK primary care clinicians, the best predictor for low-reported postoperative issues when placing composite restorations was when the majority of their total posterior restorations placed were composites.^7^ Other predictors were not using liners and using sectional matrices (recommended techniques which were not commonly used, especially by NHS dentists) (Appendix 1).^7^^,^^21^ Primarily using composite was also predictive of confidence when placing sub-gingival composites alongside those commonly using RD, and being a predominantly private dentist, for example.^21^ The current NHS system, with its large relative discrepancies in remuneration, essentially incentivises the use of amalgam. It is therefore not conducive to producing dentists who can confidently and predictably use composite posteriorly. This is likely because they are not using it regularly and are therefore not improving technically, while also having limited incentives to improve.

## Reintervention

Following failure, the nature of the subsequent reintervention (ie repair, replacement, or indirect restoration, for example) is important to understand the long-term impact of the restoration on the tooth. This reintervention may in turn be subject to huge variation, making it very difficult to study and understand. Existing data on this ‘repeat restorative cycle' is sparse. A large, long-term but old and limited NHS dataset on how differing restorative interventions affect subsequent reintervention and tooth survival at the population level exists.^36^^,^^59^ More detailed, but very short-term Dutch data are also available.^23^ Neither can really compare the impact of restoring teeth with amalgam versus composite, as the use of composite was not permitted under NHS provision in posterior non-Class V cavities at the time, and the proportion of amalgam restorations placed in the Dutch data is very small, so there is high risk of indication bias.

When removing restorations of composite in comparison to amalgam, operators with varying experience all consistently took more time, removed more sound tooth structure and left more of the existing restoration, likely because it is much more difficult to see.^60^ This is one argument for repairing rather than replacing restorations where possible in an attempt to slow the restorative cycle,^61^^,^^62^ but how often this is carried out in UK primary care is uncertain. The current evidence on repair versus replacement of both materials is limited, with two Cochrane reviews yielding no eligible studies.^63^^,^^64^ The relevant studies have different indications for repair and vary in their reported outcome measures, making drawing meaningful conclusions difficult.^28^^,^^61^

## Safety

Both a thorough Canadian health technology assessment (HTA) and Cochrane review comparing amalgam and composite restorations concluded that the evidence showed no clinically important differences in the safety of amalgam compared with composite to both patients and dental personnel.^16^^,^^65^ The known risk of a localised lichenoid reaction in the mucosa adjacent to amalgam restorations is very low.^66^ The safety of the alternatives has not been thoroughly investigated, but there are multiple reports of resin allergy involving patients and dental personnel.^9^^,^^67^ There are also health concerns surrounding some of the monomers used in composite, for example bisphenol A, and inhalation and ingestion of microplastics.^9^

## Lessons from other countries phasing-out amalgam

A United Nations Environment Programme (UNEP) document on *Lessons from countries phasing down dental amalgam use*^4^ drew heavily on a 2012 review following the phase-out of amalgam in Norway.^67^ It, alongside follow up-research, reported that the phase-out was generally well-accepted, as amalgam use was low before the ban, but there were increased costs associated with the phase-out, which were generally related to increased time required to place restorations and their more frequent replacement.^67^^,^^68^ These increases were generally borne by adult patients and were 33-50% higher for composite compared to amalgam, which was an average increase of €51 per filling for all fillings at that time.^67^ These increases in fees alone over ten years ago are comfortably higher than the current cost of any NHS direct restoration in Scotland, for example.^69^

## Costs

Posterior composite restorations take longer and are more expensive than amalgam in nearly all health systems for funders and patients.^7^^,^^48^^,^^65^^,^^67^^,^^69^^,^^70^ An exception is the NHS in England and Wales, where they cost the same.^71^ They are therefore only more expensive for the clinician in time and material costs. This essentially disincentivises their use.

Remuneration for NHS dental provision is considerably lower than in the rest of Europe. It is very difficult to compare the fee received for a single posterior restoration in England and Wales with other countries because of the unit of dental activity (UDA) system introduced in 2006; therefore, comparing a course of treatment is more appropriate. A study published in 2019 involved a questionnaire being sent to oral health policymakers in 12 European countries.^72^ It outlined a course of treatment, including two restorations, one a simple posterior restoration, with some preventive advice and scaling. Questions were then asked about the costs. The fee paid to the dentist for the course of treatment in England was €72, and in Scotland was €123.60. The fees in the other countries ranged from €158-603, with an average of €307, which equates to over four times the English fee. Though new bands to the UDA system and a minimum UDA value have recently been introduced, aiming to improve remuneration and retention of dentists within the service, the treatment plan described would still fall under the same UDA banding and therefore the fee received would not be significantly different.^73^

The differences in composite use before the ban, health service structure and costs make it very difficult to translate lessons described in the UNEP document to the current NHS system.

## Economic evaluations on amalgam versus composite restorations

Clinical outcome data and costs have been used to economically evaluate different restorative interventions. Economic evaluations (EEs) can be based solely on data gathered from a clinical trial for the period of the trial,^45^ or look to extrapolate findings over a lifetime using modelling techniques.^65^^,^^70^ Extrapolation attempts to reflect the differences between restorations over a lifetime but inevitably carries more uncertainty. All EEs and HTAs comparing amalgam with composite posterior restorations have shown amalgam to be more effective in terms of restoration and tooth survival (where assessed) and less costly.^45^^,^^65^^,^^67^^,^^70^^,^^74^^,^^75^^,^^76^ Models used have inevitably simplified the restorative cycle as the reintervention data are very limited.^65^ They have also used data sources to form the model and inform how restorations fail throughout the model, which are not relevant to the UK primary care perspective. For example, two very basic models assume restorations are replaced by the same restoration each time they fail with the same longevity.^65^^,^^74^ A slightly more sophisticated model assumes that all teeth receive replacement restorations before receiving root canal treatment and crowns, only after which they can be extracted.^70^ While this may broadly reflect the situation in Germany, which is the setting for the analysis, this does not reflect the reality of UK primary care dentistry pre-2006, with many restored teeth extracted before receiving root canal treatment and many teeth with root canal treatment not receiving crowns.^35^^,^^59^ Up-to-date information on restorations placed under NHS provision in England and Wales is very limited because of the limited data recording associated with the UDA system. This makes modelling and therefore planning future dental services in the UK difficult.

## What existing economic evaluations fail to address

Previous EEs focus on survival of restorations and teeth, and while these are clearly important to all stakeholders, composite and amalgam restorations vary in other ways which are important to UK patients, clinicians and therefore, potentially, funders.

## Patient perspectives

There are clear aesthetic benefits to composite, and data from a discrete choice experiment showed a representative sample of the UK population were willing to pay, on average, £42 more for a white compared to a silvery-grey restoration.^47^ However, they were also willing to pay £117 to experience no postoperative pain compared with persistent pain, £49 for their restoration to survive 14 years compared to five years, £40 to reduce a six-week wait to two weeks, £16 to reduce an 80-minute appointment to 20 minutes, and £14 to have treatment by a dentist rather than a therapist, as examples. Cost was by far the most important factor when selecting a restoration, however. Most of these findings favour the use of amalgam.^47^ Considering these values when designing or changing a dental healthcare system can be critical to optimising not only patient satisfaction but also uptake of services.^77^ Intervening at an appropriate time can prevent more advanced disease. This can avoid pain, morbidity and higher treatment costs. The costs can be direct, out-of-pocket costs to the patient and funder, but can also be indirect, where affected individuals miss work, which also affect employers and general societal productivity.

Traditional EEs commonly only consider costs from a single perspective. For example, the costs to the patient of providing an NHS dental restoration are different from the clinician or funder. The indirect costs for the patient of losing productive time due to having treatment performed and travelling to and from appointments, for example, have only very occasionally been accounted for in evaluating restorations, and partially so.^65^^,^^78^

## Clinician perspectives

Failure to consider or value clinician perspectives in EEs risks patient access issues. This can result from clinicians leaving the health service, or due to the increased time demands from the implementation of an alternative treatment with a limited workforce.

Incentives matter, so dentists are likely leaving the NHS in record numbers due to remuneration issues, but also a loss of trust in the NHS after the implementation of the new contract.^79^^,^^80^ This has already created an access problem for patients.^81^^,^^82^ Composite takes longer to place, longer to replace, and likely requires more frequent replacement than amalgam. Composite material costs are also currently higher for clinicians, though this may change following the EU amalgam ban. A large majority of UK primary care clinicians reported that an amalgam phase-out would impact on their ability to do their job, create appointment delays and lead to the need for more indirect restorations and extractions.^21^ An amalgam phase-out would therefore exacerbate the current access issues.

## Broader perspectives

Many of these broader costs associated with each material are not commonly considered when performing EEs, while others have been estimated. A Canadian HTA concluded that while the environmental impact of the release of mercury from amalgam was small, and amalgam separation, disposal and crematorium costs have been explored,^65^^,^^67^ the impact from composites was unknown.^65^ Other reviews have reported that mercury pollution from amalgam is a concern, however, including the Minamata Treaty.^3^^,^^83^ There are a number of potential environmental issues and therefore costs associated with composite restorations, which should be characterised.^84^

## Which patients will the phase-down and phase-out preferentially affect?

Phasing out amalgam risks preferentially impacting those with the most need in society.^6^^,^^21^ This includes low socioeconomic status groups and those with disabilities, who are all at higher risk of caries.^9^ Adequate control of the operative field to place composite may not be possible in the latter group. There is evidence of a shift in caries burden from children to adults, and with population growth and ageing populations retaining more teeth, there will be an increasing burden of caries to manage in older patients, many of whom have contributory comorbidities.^85^ Amalgam performs better in high caries-risk groups, as discussed.

In general, low-income groups value the appearance of restorations much less than higher-income groups (the difference in their average willingness to pay for a white compared to a silvery/grey filling was nearly three times lower), whereas they were willing to pay more to limit the waiting and treatment time, and cost was relatively more important.^47^ Phasing out amalgam risks access issues from both the increased clinician time required to place composite and reintervene, and the potential loss of the workforce to private practice, alongside a likely increase in patient costs. This would not provide what low socioeconomic groups value in direct restorations in the UK. It risks reducing treatment uptake, leading to more significant dental disease with increased morbidity and productivity loss, while widening already existing health inequalities.^6^^,^^21^^,^^86^

The current amalgam phase-down restricting the use of amalgam in certain groups is caveated to say ‘except when deemed strictly necessary by the dental practitioner based on the specific medical needs of the patient'.^2^ Although this is a potential solution for difficult situations, anecdotally, primary care clinicians feel placing an amalgam in children or pregnant patients carries risk for them, to which many do not wish to be exposed. The strict wording of the caveat leads to uncertainty in the consent process, the justification required and the support provided by an indemnifier should a complaint arise, alongside fear of the regulator and legal repercussions, which make it much simpler and safer for clinicians to disregard the caveat and treat the regulation as an unmitigated ban. This undermines a shared decision-making process, which should be at the heart of clinical dentistry as promoted by the FDI.^9^ It clearly affects patients, especially high caries-risk children, in whom cooperation can be limited and there is clear evidence of clinical benefit for amalgam over composite.

## Future goals

The minimal intervention (MI) philosophy is rational, and a cavity-free future of perfect prevention rendering restoration unnecessary should be the ultimate goal. This would hugely reduce the impact of any restorative material phase-out. Prevention under the MI banner is the focus of the Department of Health and Social Care's policy paper *National plan to phase down use of dental amalgam in England*.^87^ The MI philosophy is then expanded in a seemingly rational way to favour the use of composite through focusing on its ability to adhere to tooth structure which allows more minimal tooth preparations.^11^^,^^37^ It is also tooth-coloured, which is one element of a restoration that patients prefer. However, when these rational abstractions are made to face the empirical reality of current untreated caries prevalence,^88^ quality clinical data, EEs, patient preference data, UK clinician-reported data, and healthcare system constraints, all of which generally favour the use of amalgam, it does seem to fall apart somewhat. Wahl captured this well in his article titled ‘The ugly facts on dental amalgam' with a quote subtitle: ‘the great tragedy of science: the slaying of a beautiful hypothesis by an ugly fact'.^14^^,^^89^

Amalgam alternatives need to improve and their environmental impact needs to be characterised. Postgraduate composite education is not generally making clinicians confident when faced with difficult situations and needs to improve. Expert consensus on the use of techniques for restoring different cavity presentations with composite would be beneficial in guiding this, while also considering how it can be more effectively disseminated. Existing economic evaluations use rudimentary models and fail to consider clinicians and patients. They are therefore likely to significantly underestimate the broader costs of placing composite compared to amalgam over a lifetime. The current UDA system provides very limited data on restorations performed to plan future healthcare provision. The NHS dental service ideally needs to clearly define its goals. Following a consideration of its budgetary constraints, it could then design a service which incentivises the achievement of these goals while minimising unintended consequences.^90^

There are benefits to eliminating amalgam from clinical dentistry, but there are also considerable costs, and being explicit as to what those currently are is important in focusing our collective attention on ways to address the problems and sustainably plan future healthcare provision.

## Conclusions

The long-term oral health implications of an amalgam phase-out are complex to understand. However, amalgam is a simpler, quicker and more cost-effective material to place and replace than composite, which is currently the main alternative. It also has fewer postoperative complications in UK primary care, which is highly valued by the UK population. Composite restorations can be effective in difficult situations with extensive cavities, but they require high levels of technical skill and the use of expensive and time-consuming specialised equipment. These are not commonly being used in UK primary care, especially by NHS dentists. NHS remuneration for clinicians is significantly lower than in the rest of Europe. The NHS system, by therefore essentially incentivising the use of amalgam, and also disincentivising the use of recommended expensive and time-consuming equipment for composite, is likely contributing to a failure of dentists to upskill and therefore be confident in providing posterior composite restorations safely. These factors, alongside a loss of trust, have led to dentists leaving the NHS, which has created access issues for patients. The most at need in society are disproportionately affected by this. An amalgam phase-out would very likely compound this issue, widening existing health inequalities while not providing restoration characteristics which the most affected patients value most. These issues must be urgently addressed to avert an oral health crisis in the UK if amalgam is phased out in the near future.

Appendix 1 Further analysis of dataset presented in previous paper Further analysis of dataset presented in:

Bailey O, Vernazza C, Stone S, Ternent L, Roche A-G, Lynch C. Amalgam phase-down part 1: UK-based posterior restorative material and technique use. *JDR Clin Trans Res* 2022; **7:** 41-49.

The study received a favourable ethical opinion from Newcastle University Research Ethics Committee (ref 7262/2018) as part of an ongoing PhD. All participants consented to participate in the study as detailed in the paper. Data will be shared upon reasonable request to the corresponding author.

**Table 1:** High advocated composite technique use by UK primary care clinician type

**Clinician type**	High* advocated composite technique use (%)			
	Sectional matrix**	Rubber dam**	No liner**	Wedge**
NHS general dentist	13	7	23	48
Mixed general dentist	20	14	30	66
Private general dentist	31	19	37	72
CDS dentist	2	9	14	44
Therapist	10	17	26	42
Key: * = ≥76% use rubber dam, no liner, wedge, ≥51% use sectional metal matrices ** = p <0.0001 (Chi^2^) NHS = National Health Service CDS = Community Dental Services

## Data Availability

Data sharing is not applicable for this article as no new data were generated for it. The data presented in the appendix is based on a dataset from another project that will be made available on reasonable request to the author.
